# Moral Intuitions *About* Stigmatizing Practices and *Feeding* Stigmatizing Practices: How Haidt’s Moral Foundations Theory Relates to Infectious Disease Stigma

**DOI:** 10.1093/phe/phad002

**Published:** 2023-03-21

**Authors:** C Damsté, K Kramer

**Affiliations:** Faculty of Humanities, Utrecht University, The Netherlands; Faculty of Veterinary Medicine, Department of Population Health Sciences, Animals in Science and Society, Utrecht University, The Netherlands

## Abstract

Despite extensive stigma mitigation efforts, infectious disease stigma remains common. So far, little attention has been paid to the moral psychology of stigmatizing practices (i.e. beliefs, attitudes, actions) rather than the experience of *being* stigmatized. Addressing the moral psychology behind stigmatizing practices seems necessary to explain the persistence of infectious disease stigma and to develop effective mitigation strategies. Our article proposes building on Jonathan Haidt’s moral foundations theory, which states that moral judgements follow from intuitions rather than conscious reasoning. Conceptual analysis was conducted to show how Haidt’s five moral foundations can be connected to (i) moral judgements about stigmatizing practices and (ii) stigmatizing practices themselves. We found that care/harm, fairness/cheating, loyalty/betrayal and sanctity/degradation intuitions can inform moral judgements about stigmatizing practices. Loyalty/betrayal and sanctity/degradation intuitions can sometimes also feed stigmatizing practices. Authority/subversion intuitions can inform moral judgements and stigmatizing practices towards people who disrespect authoritative rules meant to protect public health. Moral dumbfounding and posthoc reasoning might explain the persistence of stigmatizing practices. In conclusion, this study demonstrates the relevance of Haidt’s approach to infectious disease stigma research and mitigation strategies. We hope that this study motivates researchers to further test and assess this approach.

## Introduction

Stigma related to infectious diseases, henceforth called ‘infectious disease stigma’, negatively affects many people (Fischer *et al.*, 2019). In the past, outbreaks of, for example the plague, cholera, measles and yellow fever led communities to marginalize people who were infected or thought to be infected (Kraut, 1998; Perry & Donini-Lenhoff, 2010). HIV-related stigma is the most well-documented infectious disease stigma in contemporary society (Avert, 2019), but stigmatization has also occurred in relation to Covid-19 (Bhanot *et al.*, 2020). In spite of extensive mitigation efforts, for example through educational and awareness campaigns, infectious disease stigma remains common.

The stigma concept has been extensively investigated by various scholars, especially within psychology and sociology (Goffman, 1963; Link & Phelan, 2001). Link and Phelan have influentially construed stigma as a phenomenon rooted in social interaction, defining it as ‘the co-occurrence of (...) labeling, stereotyping, separation, status loss, and discrimination’ and further indicating that ‘for stigmatization to occur, power must be exercised’ (Link & Phelan, 2001: 363). *Labeling* relates to the social selection of a classification that is ‘affixed’ to a person or group, such as gender or skin color or having an infectious disease. These labels can be linked to a group of undesirable characteristics leading to development of *stereotypes*. Labeled persons can be placed in distinct groups, as a way to *separate* ‘us’ from ‘them’. When labeling and stereotyping occur, an associated result is general downgrading of those persons, also called *status loss*. *Discrimination* is the unfair or unjust treatment of labeled persons. The role of power in stigma is sometimes very clear, whereas it can also be easily overlooked ‘because in many instances power differences are so taken for granted as to seem unproblematic’ (Link & Phelan, 2001: 375).

Scholars have also come to understand stigma as a category of *moral* interaction and experience. Yang *et al*. argued that the notion of stigma is an essentially moral issue, as people who are stigmatized experience threats to morally relevant interests (Yang *et al.*, 2007). They argued that adding moral experience to the concept of stigma would provide new insights in understanding the behaviors of those involved, because this allows investigation of ‘what matters most’ to people who are being stigmatized (Yang *et al.*, 2007).

This article, by contrast, concerns the moral psychology of stigmatizing *practices* rather than the experience of *being* stigmatized. According to Stangl *et al.*, stigmatizing *practices* concern beliefs, attitudes and actions, whereas stigma *experiences* include lived experiences (Stangl *et al.*, 2019: 2). Practices relate to people who stigmatize and experiences relate to people who are being stigmatized. Addressing the moral psychology behind stigmatizing practices seems necessary to explain the persistence of infectious disease stigma, and will presumably yield important lessons for mitigation strategies. Our article proposes building on Jonathan Haidt’s work to investigate the moral psychology of stigmatizing practices, as a promising new route for policies and research targeting infectious disease stigma.

Investigating how people respond to *harmless taboo violations* (see box 1), Haidt and colleagues have concluded that moral judgments typically follow from fast and unreflective cognitive processes, while conscious moral reasoning mainly takes place after a moral judgement is made, also called *posthoc reasoning* (Nisbett & Wilson, 1977; Kunda, 1990; Kuhn, 1991; Perkins *et al.*, 1991; Haidt, 2001). People often struggle or fail to support their intuitive judgment regarding *harmless taboo stories* through conscious reasoning, but will maintain their judgment nonetheless—this is phenomenon is called *moral dumbfounding* (Björklund *et al.*, 2000). Haidt and colleagues have identified five *moral foundations* or types of intuitions on which moral judgments are based. First, care/harm intuitions ‘makes us sensitive to signs of suffering and need’, while ‘it makes us eschew cruelty’ (Haidt, 2012: 178). Second, intuitions of the fairness/cheating foundation make us ‘sensitive to indications that another person is likely to be a good (or bad) partner for collaboration and reciprocal altruism’ (Haidt, 2012: 178). Third, loyalty/betrayal intuitions make us ‘sensitive to signs that another person is (or is not) a team player’ (Haidt, 2012: 178). Fourth, intuitions of the authority/subversion foundation make us ‘sensitive to signs of rank or status, and to signs that other people are (or are not) behaving properly, given their position’ (Haidt, 2012: 179). Last, the sanctity/degradation foundation involve intuitions that are evolved to protect oneself against pathogens and parasites, which ‘makes it easy for us to regard some things as “untouchable”, both in a bad way (because something is so dirty or polluted we want to stay away) and in a good way (because something is so hallowed, so sacred, that we want to protect it from desecration)’ (Haidt, 2012: 173, 179). The sanctity/degradation foundation is also referred to as the ‘purity construct’ (Shweder *et al.*, 1997).

**Box 1 – Harmless taboo violations**

In one of his most famous investigations, Haidt asked people for responses to short stories (Haidt, 2001). These stories, which were called harmless taboo violations, describe actions that many people find offensive but that do not harm anyone. An example of such a story is about Julie and Mark (see below). After hearing this story, most people immediately say that it was wrong for Julie and Mark to make love. Then, after giving their judgement, they start searching for reasons (Haidt, 2001). Haidt calls this post hoc reasoning. This happened for the story of Julie and Mark, but also for many other harmless taboo violations stories integrating with Haidt’s proposed moral foundations. From Haidt (2001), this is the story of Julie and Mark:

“Julie and Mark are brother and sister. They are traveling together in France on summer vacation from college. One night they are staying alone in a cabin near the beach. They decide that it would be interesting and fun if they tried making love. At the very least it would be a new experience for each of them. Julie was already taking birth control pills, but Mark uses a condom too, just to be safe. They both enjoy making love, but they decide not to do it again. They keep that night as a special secret, which makes them feel even closer to each other. What do you think about that? Was it OK for them to make love?” (p. 814)

In this article, we propose that stigmatizing practices in relation to infectious diseases and moral judgments about such stigmatizing practices may well be influenced by the same cognitive process and moral foundations that Haidt and colleagues have identified. As stigmatizing practices include subjective states related to judgements, such as beliefs and attitudes (Stangl *et al.*, 2019), and as Haidt argues that moral judgements are primarily based on intuitions (Haidt, 2001, 2012), it makes sense to expect that both are related. We show how Haidt’s five moral foundations can be conceptually connected to moral judgements about stigmatizing practices regarding infectious diseases and to such stigmatizing practices themselves. We enrich this conceptual analysis with examples from our previous work (Damsté & Kramer, 2021), in which we asked seven people to respond to our constructed stories that resembled Haidt’s *harmless taboo violations* but explicitly addressed infectious diseases and stigmatization. We thus argue that moral intuitions can be expected to inform moral judgements about stigmatizing practices, and sometimes even to feed those practices themselves, and illustrate how concepts from Haidt’s moral foundations theory may apply in particular cases.

## Methods

As a first step, we have mapped conceptual connections between infectious disease stigmatization on the one hand and Haidt’s five moral foundation on the other. This explorative desk research considered how the concepts defining each moral foundation (care/harm, fairness/cheating, loyalty/betrayal, authority/subversion, sanctity/degradation) relate to concepts associated with infectious diseases and stigmatization. Our analysis shows how the five foundations are *a priori* likely to (i) inform moral judgements about stigmatizing practices related to infectious diseases, and to (ii) feed infectious disease stigma. This helps to construct stories that can be used (similarly to Haidt’s *harmless taboo stories*) to trigger peoples’ moral intuitions with respect to this topic.

Second, we have illustrated our analysis with examples from previous pilot research (Damsté & Kramer, 2021), in which we asked seven interviewees to respond to six constructed stories that resembled Haidt’s *harmless taboo violations* but explicitly addressed HIV and the Covid-19 stigmatization (see Supplementary material 1). The analogy between Haidt and our constructed stories was that both concerned morally salient actions but excluded *ex hypothesi* that those actions would harm anyone. This way, both types of stories help to explore a broader spectrum of moral intuitions than care/harm intuitions only. The limitations of this earlier study cannot be overstated and we do not make any empirical claims on how intuitions are based on the five foundations inform stigmatizing practices. The fragments we have drawn from the interviews merely serve to illustrate how Haidt’s five moral foundations can sometimes be recognized in peoples’ conversations regarding stigma and infectious diseases. We hope that this will motivate adequately resourced researchers to build on Haidt’s work to study infectious disease stigma empirically.

For our earlier study, a posthoc ethical approval was given by the Faculty Ethics assessment Committee of the Faculty of Humanities (reference number: 22-161-01). The study did not pose any credible physical, psychological, social or economic risks to our interviewees and data management and informed consent procedures met generally accepted standards.

## Findings: how Haidt’s moral foundations can be conceptually linked to moral judgements about stigmatizing practices and stigmatizing practices themselves

In this section, we demonstrate how Haidt’s moral foundations can be conceptually linked to moral judgements and stigmatizing practices in the context of infectious diseases. We elaborate on each foundation and close with the phenomenon of moral dumbfounding. Table 1 presents an overview of how the foundations can be conceptually linked to judging about stigmatizing practices and stigmatizing practices themselves.

**Table 1.** Conceptual examples of intuitions that inform moral judgements about stigmatizing practices (i.e. beliefs, attitudes, actions) and intuitions that feed stigmatizing practices per moral foundation

Moral foundation	Examples that intuitions inform moral judgements about stigmatizing practices	Examples that intuitions feed stigmatizing practices
Care/harm	Not providing someone in need care, because she has an infectious disease, is wrong	-
Fairness/cheating	Not hiring someone because of her infectious disease, when she would pose no significant transmission risk to her colleagues, clients, or others, is unfair	-
Loyalty/betrayal	Not caring for people in your ingroup because they have an infectious disease is disloyal; Banishing an ingroup members that have an infectious disease is wrongful	Members from one’s ingroup should be protected from people that could transmit infectious diseases to them, by shutting such people out of the ingroup
Authority/subversion^a^	Not following up rules that authorities have established to protect public health, such as to repress an infectious disease pandemic, is wrong	Someone who does not adhere to rules meant to protect public health and control transmission of infectious diseases is labeled and discriminated for this
Sanctity/degradation	Sticking of the label ‘dangerous because of potential transmission’, in case when there is no transmission risk, is unfair	Someone with an infectious disease is labeled as polluted, impure; Not engaging in a (sexual) relationship because of the infectious disease, even if it cannot be transmitted anymore, just to be on the safe side

^a^Within this foundation, the trigger for (moral judgements about) stigmatizing practices is different compared with the other foundations: it is about non-adherence to authoritative rules related to infectious diseases, rather than to the infectious disease itself.

To enrich this conceptual analysis and to illustrate the conceptual connection between Haidt’s foundations and stigmatizing practices, we show how different intuitions can be recognized in sample fragments from pilot interviews. These fragments sometimes feature stigmatizing components, including labeling, stereotyping, separation, status loss and/or discrimination (Link & Phelan, 2001).

### The Care/Harm Foundation

The care/harm foundation makes us sensitive to signs of suffering and need, and the concepts of suffering and need connect to infectious diseases in clear ways. Care/harm intuitions will presumably trigger feelings of care for someone who is (seriously) ill because of an infectious disease, or is perceived to suffer from some other type of harm due to the infection, including mental stress due to stigmatization. It makes us share in the pain of others and to wish to avoid harm for them. These intuitions can thus give rise to moral judgements that you should care for someone in need. ‘Need’ is a broad concept and can vary from the need for care to the need for food, which the fragment below refers to:

“If she is housebound at some point, due to the [Covid-19] quarantine, then I think you should give help [bring food] if you can. Yes. If you know about that.”

An infectious disease is contagious and transmittable in nature, although under certain circumstances it can also be *untransmittable*. If there is a transmission risk, care/harm intuitions might weigh more heavily in moral judgements, as there is a potential harm to prevent to others. But if there is no transmission risk, there is no potential harm (i.e. transmitting or contracting the infectious disease) to prevent, and one would expect to see this reflected in intuitions and judgements about how to treat people carrying infections. (As explained below, however, this expectation may well be frustrated by the persistence of sanctity/degradation intuitions.)

When there is a transmission risk, this foundation could well give rise to an urge to prevent further transmission of infectious diseases, given the potential *harm* of transmission involved (of note: this can also be related to the loyalty/betrayal foundation, which is further discussed below). If someone poses a transmission risk to another, this might evoke moral judgements about risky behavior and that this person does not care for others (i.e. does not prevent transmission) which is a wrongful act. As care/harm intuitions are focused to care for the other, rather than oneself, this foundation would not provoke judgements about an urge to protect oneself (of note: the urge to prevent transmission to oneself does come back with sanctity/degradation intuitions, as discussed below).

“I think that Tom just shouldn’t take that risk [of visiting the birthday of his grandparents with Covid-19-related complaints], because they just really… If he transmits something, then he will have to carry that burden for the rest of his life. I: in what way? R: well, I think that, if you, if you do something like that and it goes wrong, then it’s something which poses a heavy burden on you because you proved that, you did harm to your grandparents, and perhaps even caused their deaths. So that seems to me, seems clear to me that you just shouldn’t take that risk.”

However, in cases where there is no transmission risk, one would expect care/harm intuitions to be more focused on the already existing harm of having an infectious disease for others, and the associated feelings of caring for or compassion for someone’s situation. Someone who refrains from offering someone care because that person has an infectious disease may well be judged as acting wrongly on these grounds (especially if providing care would not expose the caregiver to significant (transmission) risks). Not offering care can in such a case be considered a form of discrimination, prompted by the person’s ‘label’ as someone having an infectious disease, and thus as a stigmatizing practice. This suggests that care/harm intuitions can inform moral judgements about stigmatizing practices, although they do not necessarily feed stigmatizing practices themselves.

“If you can’t communicate at distance, it [caring for someone with Covid-19] is more difficult I have to say, because then you would have to ring the bell, to really converse with each other, and I don’t know if I would feel completely comfortable with that.”

### The Fairness/Cheating Foundation

The fairness/cheating foundation pertains to reciprocal altruism. It makes us want to punish cheaters, who do not stand for mutual collaboration and who are thus ‘bad collaborators’. It suppresses free riding (i.e. receiving the benefits of cooperation without carrying the costs). Someone who has an infectious disease but acts carelessly (i.e. posing transmission risks), whereas others do act in a careful way toward this person, might be judged as a bad partner for collaboration.

“If he highly appreciated that his friend cancelled the appointment the previous time [due to having Covid-19-related symptoms], then it seems to me that you should do the same. (…) If you did appreciate that your friend does so [cancelling the appointment], but you yourself prefer your own advantage and want to have fun, well, I don’t think that’s a good thing.”

Moreover, the fairness/cheating foundation might trigger intuitions and moral judgements about unequal rights, for example in case of not hiring someone on the basis of an HIV-positive status, which seems unfair if hiring the applicant would not pose a significant health risk to her prospective colleagues, customers, etc. The example below shows that fairness/cheating intuitions inform moral judgements and create certain awareness that stigma is involved (i.e. saying that it is discrimination to not hire someone with the label ‘having HIV’). This shows that fairness/cheating intuitions can inform judgements about stigmatizing practices, even though these intuitions do not necessarily lead to those practices themselves.

“My first feeling is that it [not hiring due to HIV] is simply not allowed at all, that it’s discrimination. I: and what makes it discrimination? R: well, it’s just like you won’t hire a women because of her wish to have a child.”

Another perspective is that fairness/cheating intuitions might contribute to being aware of structural forms of stigma. A similar case as argued by Henderson *et al*. on substance misuse could be made here (Henderson & Dressler, 2019): strong disapproval of inequality might result in recognition of structural processes that lead to contracting infectious diseases (such as increased HIV risk among trafficked women (Yu *et al.*, 2021) or increased Covid-19 risk among economically disadvantaged groups (Patel *et al.*, 2020)), which could be associated with thinking that having an infectious disease is not fully a personal choice. In other words, not everyone has equal chances of contracting a certain infectious disease. These intuitions might thus make people sensitive for signs of inequality in contracting infectious diseases, which relates to being aware of structural stigma.

### The Loyalty/Betrayal Foundation

Loyalty/betrayal intuitions can be expected to arise when the infectious disease influences relationships. Next to the care/harm intuitions as discussed above, the urge to prevent further transmission of infectious diseases might also be fed by loyalty/betrayal intuitions. As preventing transmission is a form of caring for another, one might say that preventing transmission towards others of your ingroup (e.g. family, friends) is part of loyalty within your relationships. This suggests that loyalty/betrayal intuitions can be a motivation for stigmatizing practices toward people outside of one’s ingroup: the urge to loyally protect friends and family can easily lead to separation from people labeled as risky for one’s ingroup. In the early Covid-19-pandemic times, for example households or small groups of friends agreed to only see each other, to limit cross transmission and extra contacts from outside, in order to create a ‘safe bubble’ with each other. Sometimes, this occurrence went so far that people were labeled as ‘safe’ (e.g. the ones who strictly adhere to the Covid-19 rules) or ‘unsafe’ (e.g. the ones who did see others and were loose with the Covid-19 rules)—and that preference was given to only let in the ‘safe’ people within ones bubble.

Second, when you refrain from providing close relatives due care, this might be considered as an act of disloyalty. An extreme example of separation is banishment of ingroup members such as friends or family, due to a person’s infectious disease. Loyalty/betrayal intuitions can help someone to recognize that such practices are stigmatizing and morally problematic.

“You can’t blame her for what happened to her, can you? I mean, if your child contracted a disease which, well… Then it’s simply just bad for her. Then you should even love her more, so to speak, be extra concerned, especially in such times close her in your arms, and not sending away. Those are things I just cannot imagine.”

In sum, loyalty/betrayal intuitions can inform moral judgements about stigmatizing practices toward people in one’s ingroup. On the other hand, loyalty/betrayal intuitions can probably lead to stigmatizing people who are labeled as a risk to one’s ingroup.

### The Authority/Subversion Foundation

The authority/subversion foundation concerns respect for authority and status and will presumably trigger moral judgements about, e.g. people who do not adhere to the Covid-19 rules and thereby show a lack of respect for authority. Stigmatization does not occur here because the person stigmatized is (suspected of) having an infectious disease, but due to their (perceived) nonadherence to rules meant to protect public health and to contain the transmission of infectious diseases. Such behavior might trigger the authority/subversion foundation and can hence lead to dismissive moral intuitions. These intuitions might in turn motivate stigmatizing practices, for example labeling and stereotyping the person as a ‘wappie’ (a Dutch neologism from Covid-19 times that roughly means ‘a weirdo who is out of touch with reality’), which implies a loss of status and invites separation and discrimination. Although the trigger for stigmatizing practices is different on this moral foundation compared with the other foundations (namely, the perception that someone subverts authoritative rules related to infectious disease control and public health protection rather than (the suspicion) that someone carriers an infectious disease), it can motivate stigmatization *in relation to* infectious diseases.

### The Sanctity/Degradation Foundation

At last, the sanctity/degradation foundation can be expected to trigger intuitions about infectious diseases or people with an infectious disease, simply because of the infectious nature or related characteristics of, e.g. how such an infection is (assumed to be) contracted. Having an infectious disease may be labeled as being ‘impure’ as it is something from outside interrupting the sanctity of the body. When this foundation is triggered, this is likely to result in negative judgements (Haidt, 2012). For example, if the infectious disease is obtained through unsafe sex or unsafe needle use, this could trigger judgements that these acts have desecrated the body. On the other hand, if the infectious disease is obtained through bad luck, this might not trigger this foundation.

In addition, sanctity/degradation intuitions may make people want to stay away from people who are potential sources of infection, even when there is no actual transmission risk, just to be on the safe side. For example, sanctity/degradation intuitions could explain why people might still feel unsure about entering into a sexual relationship with someone who has a positive HIV-status but cannot transmit the virus because effective treatment (Eisinger *et al.*, 2019):

“I would not engage so quickly in a sexual contact with a person who has HIV. But suppose it happens, sexual contact, I would just use condoms then, even if there is perhaps no risk anymore, but just to be sure. I: and why? Because you say, I would use it, even when the risk is not there anymore, but to be sure. How does that work in your mind? R7: yes stupid, yes it’s a bit crazy actually. (…) On the one hand I would say, do what makes you feel good, and use condoms or don’t use it, whatever you feel comfortable with. On the other hand, I keep having a bit of a dirty feeling in my mind then, that it might be transmitted one day. Yes, it’s a bit of a crazy feeling what is in my mind or something, can’t really explain it either.”

Avoidance due to perceived transmission risks, while there are no such risks, might be judged as avoidance on wrong grounds. Examples might be not engaging in a sexual relationship with someone with HIV but who is under effective treatment or keeping additional distance while someone is already recovered from Covid-19 and cannot transmit anymore. The act in itself can be considered as a stigmatizing practice—the label of ‘dangerous because of potential transmission’ sticks unjustifiably and leads to separation. This shows that sanctity/degradation intuitions can inform moral judgements about stigmatizing practices as well as underlie stigmatizing practices.

## Moral Dumbfounding and Post-hoc Reasoning: When Intuitive Judgements and Conscious Reasoning do not Agree and How This Might Maintain Stigmatizing Practices

Moral dumbfounding occurs when people struggle or fail to support their intuitive judgement but will maintain their judgement nonetheless (Björklund *et al.*, 2000). This might explain why people maintain to stigmatizing practices (i.e. beliefs, attitudes, actions), even when they have access to persuasive arguments that disprove their intuitive judgements leading to stigmatizing practices. The fragment below exemplifies how dumbfounding can occur within the context of infectious diseases and how it can feed stigmatizing practices. Although more research is required and we do not intend to make any substantive claims here, we hope that other researchers will be motivated to explore this further.

“Yes that person then indicates that he has it [HIV], yes God, what kind of risks does that

entail, does it have impact on the health of for example me, or well, what is the risk that I

might get sick. That I can get it [HIV]. Even though it is treatable and it is said that it cannot

be transmitted anymore, but well you don’t know that. I: what don’t you know? R: that it is not transmittable. I: but that’s what I just said. R4: yes [elongated yes], that’s what you say. [laughs] I: but what is it then, why do you think this? R: well, I don’t know. It’s something of uncharted territory. I: and what if that whole territory is known? R: yes, there is always a risk, everywhere is a risk. See, when I get a little shot soon [Covid-19 vaccine], then I can still get it, it isn’t 100% sure that I can’t get it. Nothing can be ever ruled out with 100% certainty. So even if it is well treatable, then there is always a risk, always a chance, that you still can get it. I: and suppose, very hypothetically, it is not, there is no risk, there is no chance. R: well look, it never just goes wrong because of the disease of course, but. I: what do you mean? R: well, see, a relationship with someone is not only about a disease, it’s also about how the person is, if you can get along together, there are many things. I: and if that’s also all okay? R: yes why not? Yes. But yes, that is, but yes. Well, in principle, then it is, then it could be. I: and would you feel comfortable with it, with such a relationship? R: [silence] I think so yes. Hypothetically speaking. [laughs] I: yes? R: well, you just say, if everything, if you can’t get it. Then nothing is wrong, right. I: okay, and you have to laugh now, does it really feel so? R: well, that’s so, if it really isn’t... But in my opinion, there is always a bit of ambiguity, and I think that would always gnaw a bit.”

In this fragment, the respondent expresses doubts about engaging in a (sexual) relationship due to the risks of transmission, and for the most part judges that it would be better not to have such a relationship. When the person gets access to rational arguments that HIV is untransmittable due to effective treatment, extensive posthoc reasoning occurs, in which the respondent seeks to support his judgment by offering reasons to doubt the effectiveness of the antiviral treatment. This suggests that the respondent’s judgment is based on sanctity/degradation intuitions rather than (only) care/harm intuitions, but is rationalized in terms of harm. Even when the respondent accepts (at least for the sake of argument) that there is no risk of transmission, the judgment that it would be better to avoid having this type of relationship does not fully disappear. This is arguably an example of moral dumbfounding, where an intuitive moral judgment is maintained (by persisting sanctity/degradation intuitions) even though arguments for that judgment fail. The fragment provided in the previous subsection (see page 13) provides another example of someone struggling to rationalize their intuitive judgement, and even self-consciously admitting that they lack a ‘real’ explanation, without relinquishing that judgment.

## Discussion

To the best of our knowledge, this is the first study that examines whether and how Haidt’s theory on moral intuitions can be conceptually linked to stigmatizing practices related to infectious diseases. We conducted desk research including a conceptual analysis. Our findings showed that Haidt’s moral foundations, in the context of infectious diseases, can be conceptually connected to moral judgements about stigmatizing practices and sometimes to stigmatizing practices themselves. We enriched this conceptual analysis with examples from previous work. It demonstrated the potential of applying Haidt’s moral foundations theory to infectious disease stigma, and thereby offers an innovative perspective to respond to stigmatizing practices around infectious diseases.

Our analysis suggest that care/harm, fairness/cheating, loyalty/betrayal, and sanctity/degradation intuitions indeed have the potential to inform moral judgements about stigmatizing practices regarding infectious diseases. Authority/subversion intuitions might inform moral judgments about and stigmatizing practices toward people who are perceived as subverting authoritative rules meant to protect public health and control transmission of infectious diseases. Moreover, the conceptual analysis suggests that sanctity/degradation and loyalty/betrayal intuitions can feed stigmatizing practices toward people who are (suspected of) carrying an infectious disease. This is partly in line with a study on substance use disorder. Henderson *et al.* (2019) showed that the sanctity/degradation foundation is a predictor of stigma, in which attributed stigma towards individuals even further increased when the purity construct (i.e. sanctity/degradation foundation) was triggered within a *moral* multiple regression model of substance misuse (Henderson & Dressler, 2019). In contrast with our analysis, Henderson & Dressler also concluded that the fairness/cheating foundation is such a predictor of stigma (Henderson & Dressler, 2019), although within a slightly different meaning: ‘persons who expressed a commitment to greater fairness were less likely to stigmatize, while those who emphasized purity in moral judgments were more likely to stigmatize’ (Henderson & Dressler, 2019: 16). This does not contradict our finding that fairness/cheating intuitions can inform moral judgements about stigmatizing practices rather than feed stigmatizing practices.

Nevertheless, empirical investigation is needed first to assess our conceptual analysis and to further test the potential of our approach. It is necessary to draw firmer conclusions concerning the influence of moral intuitions on moral judgements and stigmatizing practices within the context of infectious diseases in order to make substantive claims about the full ability of this approach. We hope that our conceptual analysis motivates adequately resourced researchers to build on Haidt’s work to empirically examine stigmatizing practices related to infectious diseases. Such research can draw on our conceptual analysis to construct additional stories related to infectious disease stigma, which would give more insight into the role of moral intuitions in stigmatizing practices in a wealth of situations. Within these further investigations, possible limitations of the moral foundations theory should also be considered. One critique is that the sanctity/degradation foundation is heterogenous: it has been argued that the construct lacks a coherent understanding (Gray *et al.*, 2022). Others have questioned Haidt’s theory for (more or less completely) rejecting the role of rational reasoning in moral judgement formation, rather than acknowledging the role of rationality as well as intuition in making moral judgements (Saltzstein & Kasachkoff, 2004). This article focused on exploring what resources Haidt’s approach offers for research on infectious disease stigma, but further research should also consider possible readjustments of Haidt’s approach.

Although our conceptual findings have to be first empirically investigated, they suggest that stigmatizing practices might not be completely countered by rational persuasion. Our findings showed that the phenomenon of moral dumbfounding might explain why stigmatizing practices are so persistent in spite of extensive mitigation efforts. Even when people are provided with persuasive information that confute the underlying feeling (i.e. transmission risks), stigmatizing beliefs and attitudes that are based on that feeling remain (i.e. belief of unsafety, action of not engaging in a (sexual) relationship, both due to the ‘label’ of having HIV in this specific example). We suggest that the sanctity/degradation intuitions have a persisting influence in this. This is one more reason why we hope that the relevance of Haidt’s work for infectious disease stigma will gain more recognition. Importantly, more understanding is needed about why moral dumbfounding occurs and what the exact role and function of sanctity/degradation intuitions is within this phenomenon. As Haidt noted, sanctity/degradation intuitions originally evolved to protect oneself against pathogens and parasites (Haidt, 2012: 173). Some scholars have argued, similarly, that stigmatization has an evolutionary function as it helps to prevent transmission of infectious diseases (Kurzban & Leary, 2001). However, others have argued that stigmatizing practices no longer function to protect for human communities against infectious diseases or increase the ability of modern societies to survive infectious diseases, but instead only contribute to failures in protecting public health and the health of (stigmatized) individuals (Smith & Hughes, 2014). More research is required to better understand whether the persistence of stigmatizing practices is indeed due to moral dumbfounding, what the role of sanctity/degradation intuitions is within this phenomenon, and to what extent such intuitions are ‘hardwired’ into our brains because of the supposed evolutionarily function of stigmatization in relation to infectious disease stigma.

This study has potential for program implementers, policy makers, and researchers who aim to mitigate stigma. It namely suggests that recognizing the existence of intuitions, next to conventional rational approaches (e.g. education campaigns), could contribute to more effective stigma reduction. As Haidt suggests in his *social intuitionist model*, intuitions can be (trans)formed through social interaction between people who bring different perspectives and intuitions to the table (Haidt, 2001). How the potential of social interaction to influence intuitions can be marshaled to address stigmatizing practices (i.e. beliefs, attitudes, actions) around infectious diseases deserves further study.

To use these insights about intuitions in policies addressing stigmatization, however, it is also necessary to examine which intuitions would be ethically desirable to change. According to ethical intuitionism, intuition is indispensable in making (justified) ethical judgments, although it remains possible to reflect on and reject particular intuitions (e.g. (Roeser, 2010)). It may be that some intuitions with respect to infectious disease stigma (e.g. the intuition that infected persons should be shunned even if there is no risk of transmission) are on reflection ethically problematic, while others (e.g. the intuition that not hiring someone for a desk job because she is HIV-positive is unfair) deserve to be fostered. Future ethical analysis is required to investigate to what extent and under what conditions moral intuitions feeding stigmatizing practices related to infectious diseases are morally (un)justifiable. Such an ethical analysis should draw on empirical research regarding the intuitions that actually feed stigmatizing practices, however, and this study suggests that Haidt’s moral foundations approach offers a promising new route for such research.

## Supplementary Material

phad002_suppl_Supplementary_Materials

## About the authors

Carlijn Damsté has a BSc and MSc in Biomedical Sciences and a MA in Applied Ethics. Dr. Koen Kramer is a postdoctoral researcher and lecturer in ethics. This publication is the result of the thesis that Carlijn Damsté wrote during her MA degree, supervised by Dr. Koen Kramer.
