# Correction to: Moral Intuitions *About* Stigmatizing Practices and *Feeding* Stigmatizing Practices: How Haidt’s Moral Foundations Theory Relates to Infectious Disease Stigma

**DOI:** 10.1093/phe/phad009

**Published:** 2023-04-08

This is a correction to: Carlijn, Koen, Moral Intuitions *About* Stigmatizing Practices and *Feeding* Stigmatizing Practices: How Haidt’s Moral Foundations Theory Relates to Infectious Disease Stigma, Public Health Ethics, 2023;, phad002, https://doi.org/10.1093/phe/phad002

In the originally published version of this manuscript, ‘Box 1’ was inadvertently omitted from the Introduction section.

This error has been corrected.

**Box 1 – Harmless taboo violations**

In one of his most famous investigations, Haidt asked people for responses to short stories (Haidt, 2001). These stories, which were called harmless taboo violations, describe actions that many people find offensive but that do not harm anyone. An example of such a story is about Julie and Mark (see below). After hearing this story, most people immediately say that it was wrong for Julie and Mark to make love. Then, after giving their judgement, they start searching for reasons (Haidt, 2001). Haidt calls this post hoc reasoning. This happened for the story of Julie and Mark, but also for many other harmless taboo violations stories integrating with Haidt’s proposed moral foundations. From Haidt (2001), this is the story of Julie and Mark:

“Julie and Mark are brother and sister. They are traveling together in France on summer vacation from college. One night they are staying alone in a cabin near the beach. They decide that it would be interesting and fun if they tried making love. At the very least it would be a new experience for each of them. Julie was already taking birth control pills, but Mark uses a condom too, just to be safe. They both enjoy making love, but they decide not to do it again. They keep that night as a special secret, which makes them feel even closer to each other. What do you think about that? Was it OK for them to make love?” (p. 814)

