# Supplementary material for: Moral Intuitions About Stigmatizing Practices and Feeding Stigmatizing Practices: How Haidt’s Moral Foundations Theory Relates to Infectious Disease Stigma
Source: Public Health Ethics. 2023 Mar 21;16(1):102–11. doi: 10.1093/phe/phad002 (PMC10161519; doi:10.1093/phe/phad002)
Supplement: phad002_suppl_Supplementary_Materials [file phad002_suppl_supplementary_materials.pdf]

Moral Intuitions *About* Stigmatizing Practices and *Feeding* Stigmatizing Practices: How Haidt's Moral Foundations Theory Relates to Infectious Disease Stigma

C. (Carlijn) Damsté and K. (Koen) Kramer

Supplementary materials

Supplementary material 1: Constructed stories

Constructed stories regarding HIV

Story "Sam": Sam is HIV-positive. He is under treatment, which means that he cannot transmit his HIV anymore. He would love to have a relationship, but every time when he discloses his HIV, he is rejected. What do you think of this situation?

Story "Nina": Nina applies to a new job, and she has a good conversation with her future boss. She seems to be a perfect match for the job. Then she discloses her HIV. On the basis of her HIV, she is not hired. What do you think of this situation?

Probe question: what if Nina applies for a job in the sex industry? What do you think about that? Suppose Nina is also under good treatment, so she cannot transmit her HIV, does this matter for you and how?

Story "Julia": Julia does not disclose her HIV to her family. She thinks that she will be ostracized when she does disclose. Julia cannot transmit her HIV. What do you think of this situation?

Constructed stories regarding Covid-19

Story "Marie": Marie has contracted Covid-19, while she followed up all Covid-19 rules. She is actually quite sick of it and feels bad. Of course, she cannot go to the supermarket for groceries,

while she does not have food at home, but she also does not have someone who can do groceries for her. What do you think of this situation?

Story "Lisa": Lisa has been cured of Covid-19 for one week, so she cannot transmit the virus anymore. Still, her friends keep 2,5 meters distance instead of 1,5 meters. Besides, her friends prefer not to come by. One friend told Lisa not to come or the coming month. What do you think about this?

Story "Tom and Lars": Tom has a mild cold and sometimes he coughs, but he actually just feels well. His grandfather's 85th anniversary is coming soon, and Tom would like to congratulate his grandfather. He decides to visit his grandparents, despite of his mild cold. He keeps enough distance. What do you think of this situation?

Probe question: Suppose that Tom does not visit his grandparents, but his friend Lars. Last month, Lars also had a mild cold, which was the reason why Lars cancelled their appointment. At that moment, Tom highly appreciated the action of Lars. Now, it is exactly the same: Tom and Lars will meet again, but now Tom has a mild cold. Tom does not cancel their appointment, because he was looking forward to it. This is the opposite of what Lars did last month. They keep enough distance. What do you think of this situation?
